# Developmental Support for Sick Children through Play in Japan’s ECEC System: A Comparison with Hospital Play Specialists

**DOI:** 10.3390/children5100133

**Published:** 2018-09-21

**Authors:** Narumi Kihara, Tomoko Yamamoto

**Affiliations:** Department of Child Development, Faculty of Humanities, Saitama-Gakuen University, 1510 Kizoro, Kawaguchi 3330831, Japan; na0719ru@i.softbank.jp

**Keywords:** child, sick, ECEC, HPS, Japan

## Abstract

Early childhood education and care (ECEC) workers and hospital play specialists (HPSs) share a role in supporting the development of sick children through play while respecting their autonomy. On the other hand, in supporting children’s play, managing their anxiety, and making environmental arrangements for them, ECEC workers and HPSs play different roles. When supporting the development of sick children, the former should respect their autonomy and make the most of the characteristics of ECEC as a measure to comprehensively support their participation in play, including it in their daily lives. ECEC workers are also expected to contribute to the further development of comprehensive support by promoting collaboration and cooperation with various professionals, including HPSs.

## 1. Introduction

In Japan, early childhood education and care (ECEC) is comprehensively provided through daily life and play, in general. Some ECEC facilities also deal with sick children.

In terms of developmental support for sick children through play in Japan’s ECEC system, previous studies examined the specialty of ECEC workers in managing such children [1], the role of education and medical care in supporting their participation in play [2], appropriate play activities to be used in education and medical care [3], and ECEC workers’ awareness of play for hospitalized children [4]. Practical approaches to play were also reported [5].

Professionals supporting sick children through play include hospital play specialists (HPSs) who are actively nurtured as nationally certified professionals in the United Kingdom. In the United Kingdom, HPSs are expected to help sick children in hospitals cope with their situation through therapeutic play activities, with an understanding of child development [6]. To work as a HPS, it is necessary to obtain a basic academic degree in this field, and register with the Healthcare Play Specialist Education Trust (HPSET). The ability to establish favorable relationships with children, their parents, and care-givers, in addition to advanced communication skills, creativity, and organizational skills to plan and perform therapeutic activities are also needed. The role of HPSs in the United Kingdom involves helping children prepare for treatment, distracting their attention during surgery, and supporting them to understand this experience through play. They are also called healthcare play specialists. In Japan, an HPS training program started at the University of Shizuoka Junior College in 2007 [7]. Training is continuously being provided through specialized lectures for members of society. Similarly to those in the United Kingdom, Japanese HPSs use therapeutic play to reduce fear and pain in children and support them to face treatment [8]. At the same time, in Japan’s ECEC system, ECEC workers represented by those specializing in education and medical care for pediatric patients also support sick children’s participation in play. Child Welfare Act Article 18-4 defines ECEC workers as “professionals who provide ECEC for children and related guidance for their parents using their expertise and skills in the name of an ECEC worker”. Students who desire to become ECEC workers need to obtain the necessary credits as determined by the government at universities, junior colleges, or technical schools providing ECEC worker training courses in order to qualify for ECEC worker licensing after graduation. Subsequently, they should apply for registration by prefectural governors, and receive an ECEC worker certificate to be able to practice. ECEC workers comprehensively provide ECEC through daily life and play [9]. They create environments for children to voluntarily and willingly participate in play, and, placing importance on their autonomous activities and communication with other children, help them acquire appropriate experiences during infancy.

This paper discusses similarities and differences in support for sick children to participate in play between ECEC workers and HPSs in Japan. It also examines distinctive roles the former are expected to play, based on the results of a survey involving an HPS.

## 2. ECEC Workers’ Approaches to Play

Play is a large part of a child’s life. Children experience the joy of life, the pleasure of empathizing with others, and a sense of self-confidence through it. Sick children are not exceptional, but their capability to develop play independently even in the presence of a disease and related limitations depends on ECEC workers’ approaches.

In Japan’s ECEC system, approaches for sick children mainly through play are classified based on the pathological condition, disease, and ECEC method [10].

### 2.1. Classification Based on the Pathological Condition

Some pathological conditions require bed rest. In such cases, in-bed ECEC, mainly consisting of low-intensity or passive play activities, is provided. For children requiring rest in indoor environments, indoor ECEC, which is applied in most cases before returning to general education/care settings, is provided. As it also targets children who have markedly recovered, methods similar to normal ECEC activities such as relatively high-intensity, group, and outdoor (balcony) play, are used in some cases. When providing indoor ECEC, it is necessary to maintain an appropriate activity–rest balance by, for example, ensuring sufficient naps, similarly to the case of general ECEC. Making environmental arrangements and providing appropriate guidance for children to return to bed whenever necessary, according to their pathological conditions, are also important approaches.

### 2.2. Classification Based on the Disease

ECEC for sick children also consists of group activities to be performed in ECEC rooms. However, for those with specific diseases including influenza, individualized ECEC is provided in observation or isolated rooms. These rooms aim to prevent secondary infection in ECEC for sick children. When treating them in isolated rooms without any companions, ECEC workers should clarify their favorite activities to reduce their anxiety in the early stages. Sufficient affectionate touch for younger children, previous explanation of treatment processes for older children, and proactive statements such as “We will play outdoors when you are healed”, are regarded as effective in these cases. Furthermore, as isolated room use generally lasts for 2 days in many cases, importance is placed on ECEC planning to help children lead their daily lives with future perspectives by making statements, such as “We will play doing XXX tomorrow”, at the end of each session.

### 2.3. Classification Based on the ECEC Method

ECEC for sick children are also classified into intention-based and goal-oriented ECEC. The former is based on children’s own intentions, while the latter is planned, implemented, and reviewed based on goals set by ECEC workers. In both cases, it is necessary to respect children’s autonomy. At this point, ECEC workers are expected to create environments for children to voluntarily and actively participate in play. Play activities used in intention-based ECEC vary depending on the age, developmental stage, and pathological condition. ECEC workers should make environmental arrangements for children to develop their activities based on their interest/curiosity. When infants and preschoolers share the same ECEC room, it is necessary to organize a section exclusively for the former to crawl in and another exclusively for the latter to play in using blocks, for example. Play activities used in goal-oriented ECEC include birthday parties and seasonal events.

## 3. Practical HPS Approaches to Play

The authors examined an HPS working at a developmental rehabilitation center in Osaka City, who cooperated. Developmental rehabilitation centers where HPSs work are highly specialized hospitals, mainly treating children with motor impairment due to paralysis resulting from damage to the central nervous system. They also function as community-based support centers. We asked a highly specialized HPS, who supported children with physical and/or severe motor and intellectual disabilities through play, to cooperate with the study, and obtained her consent. The HPS mainly worked a daily shift from 08:30 to 17:30 for 39 h a week, with 8 days off every 4 weeks, in principle. She mainly worked on wards for children with physical and severe motor/intellectual disabilities with co-workers whose ages widely ranged from their twenties to their sixties.

She was interviewed and responded to a questionnaire in August 2016. We only examined the HPS, and avoided filming children from the perspective of privacy protection. We filmed some toys with her permission to use these images for research purposes. In addition to her cooperation, we also obtained the approval of her developmental rehabilitation center.

We only examined the HPS, and avoided filming children from the perspective of privacy protection. We filmed some toys with her permission to use these images for research purposes. In addition to her cooperation, we also obtained the approval of her developmental rehabilitation center.

To compare HPSs’ and ECEC workers’ approaches to play (presented in Section 2), we mainly investigated the activities of the HPS, such as ECEC contents, related considerations, examples of play, parental support, and collaboration with other professionals. We also examined her recognition of the role of HPSs and differences with respect to ECEC workers. The results are summarized as follows.

### 3.1. Children’s Age and ECEC Contents

HPSs generally deal with children from 0 to 18 years of age. The HPS examined in this study mainly dealt with about 10 children ranging from 0 to 12 years of age.

ECEC was provided for 4 days weekly; 2 days for individualized and group ECEC, respectively. ECEC plans were developed based on a daily schedule for each child (e.g., eating > dressing > playing > rehabilitation). At the beginning and end of ECEC, the same music was played each day, with a view to promoting appropriate life rhythms.

Play activities supported by HPS include those for therapeutic purposes. Therapeutic activities aim to help children cope with invasive treatment and care and become mentally stable by expressing their emotions. These activities are represented by non-instructive play. The HPS carefully observed individual children’s play and the course to confirm whether they sufficiently expressed their anxiety and fear through non-instructive play. Based on the results of observation, she presented children with play choices.

The HPS also stated that when supporting children with end-stage conditions, she focused on their lives until the end. She complied with the basic principle of respecting children’s choices when they preferred conversing to playing.

### 3.2. Consideration and Devised Approaches for Children

The HPS made arrangements to create safe and secure environments for children whose physical conditions frequently changed. Play activities for them were determined upon deliberations with the nurse in charge on each day. The HPS attentively monitored changes in children’s facial expressions, eyeball movements, and heart rates, as well as other data shown by medical devices, to accurately recognize their responses, even only slightly. She did this based on the idea that play is the easiest activity for children to express themselves. For example, when listening to music, their eyes actively move, representing their response. The HPS made commitments to these responses through active communication or other approaches.

### 3.3. Examples of Play

The HPS emphasized the significance of each play activity. In addition to picture book reading and seasonal events, she introduced slime, button-dropping, balloon clay, and play with bubbles/sand.

She made special consideration for children with visual impairments. For example, when reading a picture book about a ladybug, she pronounced more clearly, with her month largely moving, and explained that the ladybug is an insect of red color with a round shape to help children imagine this character. Based on her experience, she noted that glittering materials and the sounds of waves and music boxes are effective to reduce children’s pain.

During play activities, the HPS placed importance on the effect of touching toys. To provide all children with such opportunities, toys had been prepared, many of which were handmade.

**Figure 1:**
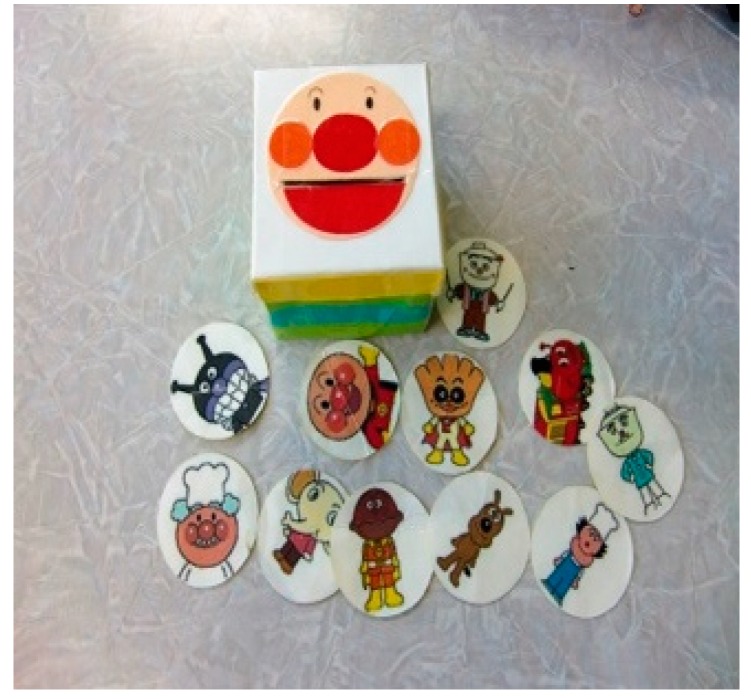
Anpanman savings box (created using a milk carton).

**Figure 2:**
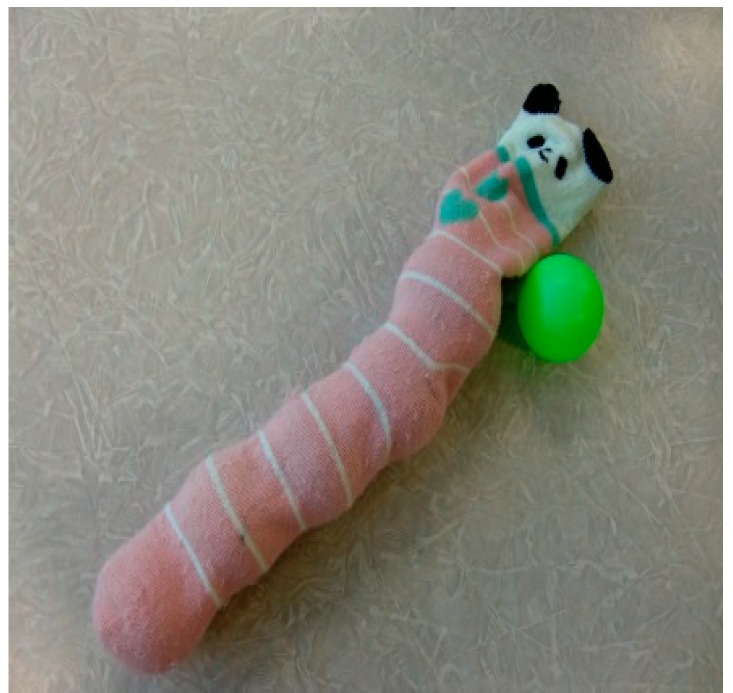
Filling a sock with balls (created using unneeded socks).

**Figure 3:**
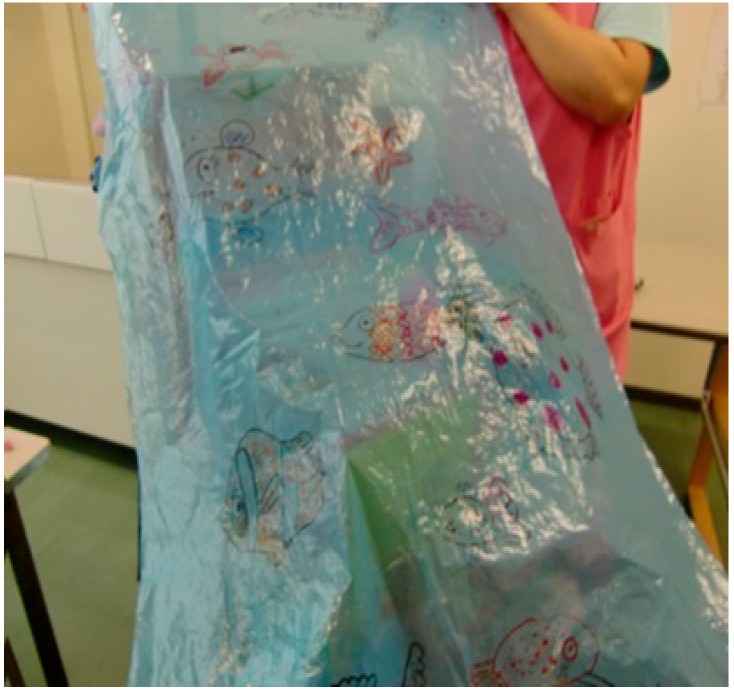
World of the sea (created using a plastic sheet).

**Figure 4:**
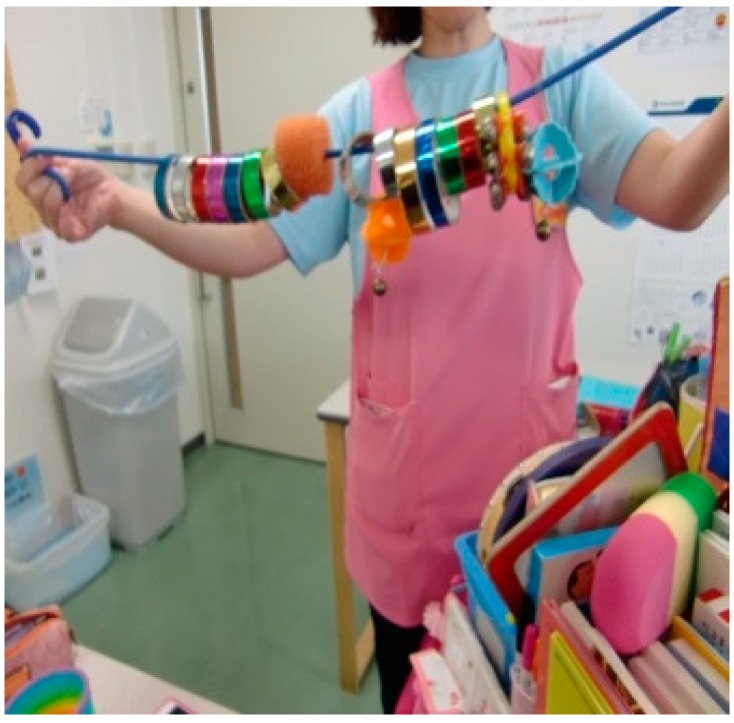
Cord and rings.

Some of the handmade toys used for play with sick children are listed below (Figure 1, Figure 2, Figure 3 and Figure 4):

Figure 1: This handmade toy is a saving box shaped like Anpanman for children to enjoy touching original coins with various Anpanman characters drawn on and dropping them into the saving box. The coins with visually enjoyable drawings are also usable as cards.

Figure 2: Children enjoy movements to fill socks with balls and take them out, as well as various sensations when touching these materials.

Figure 3: Fishes of the sea are drawn on a blue plastic sheet. It is also possible to paste fish drawings onto the sheet, or use the sheet for fishing games. With the sheet spread on the bed, children lying in bed can also enjoy the world of the sea.

Figure 4: This toy consists of a cord, hooks, and various materials with holes, such as rings made from glittering tapes of different colors and bells, threaded onto the cord. For children in bed, the cord is hooked at the bedrail, and rings are slid toward the opposite side at a signal from HPS, such as “Here they go! Choo!”, so that they enjoy the movements and sounds of rings that move while rotating and changes of color. 

To evaluate the effects of these play activities on sick children, the HPS observed changes in their gazes and facial expressions, lip and fingertip movements, and increases in their pulses, as even those with severe motor/intellectual disabilities show responses to toys and play, according to her.

The HPS continuously improved the quality of her own practice through support for children using play. In the process of support, she mainly observed whether each play activity amused and healed children, and it was appropriate for the current and future development of individual children. Furthermore, she continuously participated in a training program provided by the non-profit organization HPS Japan to improve her own skills. For example, lectures given in February 2018 as part of this program aimed to help participants improve their skills to support the development of children with various medical needs, such as those with multiple disabilities and intractable diseases, through play.

### 3.4. Parental Support

The HPS also provided parents with information regarding their children’s play. She had special consideration for parents who could not frequently visit the hospital due to living in a distant area or who worked by explaining or demonstrating the progress of their children to share their growth.

### 3.5. Collaboration with Other Professionals

The HPS recorded play sessions on each day in medical charts to share play-related information with other professionals. When meeting new children, the HPS previously discussed with other professionals and observed their support for the children to plan appropriate play activities.

### 3.6. Recognition of the Role of HPS

Supporting the development of children through cooperation with medical teams, the HPS developed a sense of accomplishment. She was pleased to see children’s positive responses when she took held their hands or swung their arms to dance together.

The HPS also made arrangements for children at the end of their lives to be able to play as much as possible mainly through relaxing activities, such as play with light or music boxes. When they faced stressful situations, the HPS gently talked to them while holding their hands, and coordinated for them to stay with their families as much as possible. When they died, she adopted measures to avoid negatively influencing other children, such as consulting her co-workers and appreciating positive aspects.

### 3.7. Differences between ECEC Workers and HPS

HPS and general ECEC workers share many roles, but the former mainly plan and organize activities for children to support their play based on their wishes, rather than using play as a measure of developmental support.

## 4. Discussion

This paper compares ECEC workers’ and HPSs’ approaches to play presented in Section 2, respectively.

Respect for children’s autonomy may be a common characteristic of developmental support for sick children through play provided by ECEC workers and HPSs in Japan.

The HPS who cooperated with the study regarded ECEC workers’ approaches as important when supporting sick children, whose capability to develop play independently even in the presence of a disease and related limitations depends on them. ECEC workers are expected to accept the emotions and wishes of children as autonomous individuals, and enable them to perform activities with a sense of security and confidence. When making environmental arrangements for them, it is necessary to allow them to participate in environments, voluntarily perform activities, and acquire various experiences based on the recognition of play that accounts for a large part of a child’s life, providing children (including those with illness) with opportunities to experience the joy of life, the pleasure of empathizing with others, and a sense of self-confidence. HPSs are expected to attentively listen to children receiving treatment, and accept their emotions/wishes in order to organize and offer environments for child-centered medicine. The HPS recognized that ECEC workers and HPSs share many roles in supporting the development of children.

On the other hand, the two types of professionals also play different roles in Japan.

First, their methods to support children’s play are different. Support from ECEC workers is characterized by comprehensive approaches to children’s play and daily lives to help them acquire appropriate experiences during childhood. Children spend most of their time in the ECEC setting during an important period for their lifelong personal development. To create environments for children to lead a healthy and emotionally stable life and fulfill themselves, importance is attached to appropriate life rhythms. The development of basic habits and attitudes necessary for daily life is also considered as a basis for mental and physical health. In contrast, HPSs support children mainly through play. Based on a play program for each child, they develop activities that are enjoyable for all children, with a view to preventing their hospital lives from being oriented only by treatment.

Second, their approaches to children’s anxiety are different. In the case of sick children, especially hospitalized children, their participation in play should be supported in consideration of their anxiety as a factor leading to changes in their conditions. In ECEC, nursing (which directly influences children’s emotional stability) and education are integrally provided. ECEC workers perform nursing to maintain children’s lives and promote their emotional stability through support and commitments. Such nursing aims to help children lead their lives with stable conditions, express their feelings with a sense of security, feel self-affirmative, and become mentally and physically healed [9]. In order to promote children’s emotional stability, it is necessary to fulfill their various needs in environments with sufficient care and a relaxing atmosphere. In contrast, play activities supported by HPSs include those for therapeutic purposes, represented by non-instructive play. Therapeutic activities aim to help children cope with invasive treatment and care, and become mentally stable by expressing their emotions. HPSs also support children to avoid negative emotions, such as fear and resignation. Another important point of support for children’s play from HPS in terms of reducing their mental burden is not to expect successful outcomes.

Third, the purposes of environmental arrangements by them are different. In ECEC, environmental arrangements are made for children to mindfully live in the present moment, and acquire basic strengths for favorable future creation. Practical ECEC approaches to such arrangements include: providing opportunities for children to acquire various experiences, creating relaxing and familiar places with a warm atmosphere for them to develop productive activities, and promoting their interpersonal communication skills to fulfill themselves through such activities [9]. In contrast, HPSs organize and offer environments for child-centered medicine. They cooperate with medical teams to support children through play. They also target children with end-stage conditions and those surrounding them. While enjoying relaxing play activities with children as much as possible, they provide special approaches, such as holding their hands and gently talking to them, according to their situations.

Based on these similarities and differences, ECEC workers supporting the development of sick children should respect their autonomy, and make the most of the characteristics of ECEC as a measure to comprehensively support their participation in play, including their daily lives. ECEC workers are also expected to contribute to the further development of comprehensive support by promoting collaboration and cooperation with various professionals, represented by HPS.

Sick children need two types of play: play to manage their sickness, such as therapeutic play activities and those according to developmental stages, and play that can be similarly undertaken by sick and healthy children for its own sake. Therefore, in order to effectively support sick children, it may be important to develop support approaches involving more diverse professionals, by further promoting collaboration and cooperation among them.

## 5. Conclusions

This paper examines distinctive roles ECEC workers are expected to play in supporting the development of sick children through play, based on the results of a survey involving an HPS.

Sick children’s mental and physical conditions tend to frequently change, possibly due to their anxiety. Similarly to the case of other children, they also experience the joy of life, pleasure of empathizing with others, and a sense of self-confidence through play. Therefore, helping them develop play independently may be a challenge of ECEC.

Professionals supporting sick children’s participation in play include HPS, in addition to ECEC workers. HPSs develop play programs for individual children in special environments, such as hospitals, with a view to preventing their hospital lives from being oriented only by treatment. The HPS who cooperated with the present study offered multiple play choices based on the developmental stage, disease, and pathological condition, with various handmade toys available.

Respect for children’s autonomy may be a common characteristic of developmental support for sick children through play provided by ECEC workers and HPSs. On the other hand, in supporting children’s play, managing their anxiety, and making environmental arrangements for them, ECEC workers and HPSs play different roles. The former are also expected to contribute to the further development of comprehensive support by promoting collaboration and cooperation with various professionals, represented by HPSs.
